# A Review of Novel Cardiac Biomarkers in Acute or Chronic Cardiovascular Diseases: The Role of Soluble ST2 (sST2), Lipoprotein-Associated Phospholipase A2 (Lp-PLA2), Myeloperoxidase (MPO), and Procalcitonin (PCT)

**DOI:** 10.1155/2021/6258865

**Published:** 2021-08-09

**Authors:** Junpei Li, Tianyu Cao, Yaping Wei, Nan Zhang, Ziyi Zhou, Zhuo Wang, Jingyi Li, Yue Zhang, Sijia Wang, Ping Wang, Nannan Cheng, Lijing Ye, Minghui Li, Yu Yu, Congcong Ding, Ziheng Tan, Biming Zhan, Qiangqiang He, Huihui Bao, Yanqing Wu, Lishun Liu, Jianping Li, Xiping Xu, Xiaoshu Cheng, Xiao Huang

**Affiliations:** ^1^Department of Cardiology, Nanchang University Second Affiliated Hospital, Nanchang, China; ^2^University of California, Santa Barbara, CA, USA; ^3^Beijing Advanced Innovation Center for Food Nutrition and Human Health, College of Food Science and Nutritional Engineering, China Agricultural University, Beijing, China; ^4^Department of Cardiology, Peking University First Hospital, Beijing, China; ^5^Graduate School at Shenzhen, Tsinghua University, Shenzhen, China; ^6^State Key Laboratory of Natural Medicines, Research Center of Biostatistics and Computational Pharmacy, China Pharmaceutical University, Nanjing, China; ^7^Public Health College, Sun Yat-Sen University, Guangzhou, China; ^8^Shenzhen Evergreen Medical Institute, Shenzhen, China; ^9^National Clinical Research Center for Kidney Disease, State Key Laboratory for Organ Failure Research, Guangdong Provincial Institute of Nephrology, Division of Nephrology, Nanfang Hospital, Southern Medical University, Guangzhou, China

## Abstract

While the received traditional predictors are still the mainstay in the diagnosis and prognosis of CVD events, increasing studies have focused on exploring the ancillary effect of biomarkers for the aspiring of precision. Under which circumstances, soluble ST2 (sST2), lipoprotein-associated phospholipase A2 (Lp-PLA2), myeloperoxidase (MPO), and procalcitonin (PCT) have recently emerged as promising markers in the field of both acute and chronic cardiovascular diseases. Existent clinical studies have demonstrated the significant associations between these markers with various CVD outcomes, which further verified the potentiality of markers in helping risk stratification and diagnostic and therapeutic work-up of patients. The current review article is aimed at illuminating the applicability of these four novels and often neglected cardiac biomarkers in common clinical scenarios, including acute myocardial infarction, acute heart failure, and chronic heart failure, especially in the emergency department. By thorough classification, combination, and discussion of biomarkers with clinical and instrumental evaluation, we hope the current study can provide insights into biomarkers and draw more attention to their importance.

## 1. Introduction

Cardiovascular disease (CVD), a major global disease burden, is the leading cause of death and disability worldwide [1]. Among these, acute myocardial infarction, acute heart failure, and chronic heart failure are the most common manifestations. There are validated biomarkers used at present, for example, troponin which possesses high specificity and sensitivity when diagnosing acute myocardial infarction and BNP/NT-proBNP which is used to assess the severity of heart failure. Although the treatment of CVD has made gratifying progress, traditional biomarkers inevitably have the problem of residual risk omission in the screening of high-risk groups and accurate diagnosis, management, and prognosis of the disease. For this reason, the studies of novel biomarkers are becoming the main task. The search for novel biomarkers with a long half-life and good stability of myocardium has a wide range of applications and values in clinical diagnosis. In the pursuing of precision, the study of biomarkers has become one of the main tasks in CVD. This article reviews four novel cardiac biomarkers which are very significant but have not been used routinely in clinical practice. These biomarkers can be expected for future application in clinical practice.

## 2. Biological Metabolism of Myocardial Markers

*Soluble suppression of tumorigenicity 2* (*sST2*) is a member of the IL-1 receptor family, which contains two subtypes, soluble ST2 (sST2) and transmembrane ST2 (ST2L), mainly expressed in cardiomyocytes, reflecting the stress of the ventricular wall and is associated with inflammation and immune response [2]. The interaction of ST2L and IL-33 can mitigate cardiac hypertrophy and myocardial fibrosis, thereby exerting protection on the myocardium. On the contrary, sST2 removes IL-33 from the circulation, promoting cardiac hypertrophy, fibrosis, and ventricular dysfunction [3].

The expression of sST2 was upregulated with myocardial ischemia or mechanical stress, which plays an important role in myocardial remodeling after ischemic injury. It is a promising prognostic biomarker for predicting future clinical heart failure and death in myocardial ischemia patients and has synergy with established heart biomarkers in predicting value [4].

Lipoprotein-associated phospholipase A2 (Lp-PLA2) belongs to group VII of the PLA2 superfamily, currently composed of six types and divided into 16 distinct groups [5]. This enzyme is secreted mainly by macrophages and circulates in the blood as a compound of low-density lipoprotein (LDL) and high-density lipoprotein (HDL). Lp-PLA2 most often combines with LDL (>80%) and HDL (round 10%), whereas it rarely interacts with VLDL or Lp (a) [5]. Lp-PLA2 can hydrolyze oxidized LDL to two biologically active products, named lysophosphatidylcholine (lysoPC) and oxidized nonesterified fatty acids (oxNEFAs). LysoPC appears to stand for most of Lp-PLA2-caused inflammation responses. It has a strong proinflammatory and proatherosclerosis effect [5]. The high upregulation of Lp-PLA2 in atherosclerosis plaques is associated with plaque rupture and may therefore represent underlying vascular inflammation and plaque rupture [6]. Results based on available basic and epidemiological studies suggest that lowering Lp-PLA2 reduces the risk of cardiovascular disease, which grants Lp-PLA2 potential to become an important diagnostic index for the risk prediction and prognosis of CVDs.

Myeloperoxidase (MPO) is a key element of the innate immune system. It is found in the aniline blue granules of myeloid cells (mainly neutrophils and monocytes) and is a specific marker of myeloid cells. During neutrophil activation, lysosomal-phagosome fusion leads to the release of MPO, while the assembly of NADPH oxidase complex on the inner surface of the membrane leads to the production of superoxide anions (O_2_^−^), which rapidly decompose to hydrogen peroxide (H_2_O_2_). MPO catalyzes the reaction of H_2_O_2_ with chloride ions (Cl^−^) to form hypochlorous acid (HOCl), which helps to destroy microorganisms in the phagocytic lysosome. However, some MPO were released extracellularly, where HOCL is produced to promote the development of host tissue damage and disease [7]. Although the development of HOCl has a crucial effect on removing pathogens, HOCl and other reactive species derived from MPO cause oxidative tissue damage and cell dysfunction, which further promote disease outcomes [8]. In addition, MPO plays an important role in atherosclerosis and cardiovascular and cerebrovascular diseases [9]. It involves the formation of atherosclerosis that is modified by LDL, reduces the antiatherosclerotic properties of HDL [10, 11], contains bioavailability of nitric oxide and leads to endothelial dysfunction [12, 13], and promotes endothelial cell apoptosis and thrombosis.

Procalcitonin (PCT) is a propeptide substance of no hormone activity calcitonin (CT). This glycoprotein is composed of 116 amino acids and has a molecular weight of 13,000. During normal metabolism, PCT, generated from thyroid C cell secretion, can rarely be detected in serum or cerebrospinal fluid in a healthy population (<0.05 *μ*g/l) [14]. However, PCT releasement upsurges due to the upregulation of endotoxins and other mediators (such as interleukin- (IL-) 1B, tumor necrosis factor- (TNF-) a, and IL-6) [15]. These characteristics made PCT preferable to markers such as white blood cell (WBC) or CRP to possess more specificity for bacterial infections and may allow PCT to be used to distinguish bacterial from viral infections and other noncommunicable diseases [16, 17]. Until now, a growing number of researchers have supported PCT testing in patients with CVDs, including patients with shortness of breath, possible heart failure, suspected endocarditis, and acute coronary syndrome [18].

## 3. Clinical Impact of Cardiac Markers

### 3.1. sST2

The American heart failure cohort study (PRIDE study) [19] which analyzed 593 patients from the emergency department with acute dyspnea (with or without HF) found that sST2 concentrations were significantly higher in ADHF patients than in non-HF patients. Although NT-proBNP was significantly superior to sST2 in diagnosing ADHF, sST2 has more important value when it comes to prognosis since the ADHF-induced mortality population, within one year, has significantly higher sST2 concentration than survivals (1.08 vs. 0.18 ng/ml, *P* < 0.001). It is noteworthy that the prognostic value of both sST2 and NT-proBNP is additive; that is, patients with increased NT-proBNP and sST2 have the highest mortality rate in 1 year, which is about 40%; the prognostic value of sST2 combined with NT-proBNP maintained 4 years after the disease onset.

The PRIDE research [20] also shown that, compared to survivors, patients who died in 1 year (67.4 vs. 35.8 ng/ml; *P* < 0.001) and 4 years (47.4 vs. 35.6 ng/ml, *P* = 0.01) have a higher sST2 level. Thus, sST2 ≥ 35 ng/ml was recommended as a predictor threshold for a poor prognosis, and the risk of death is significantly increased once beyond this threshold value. In 2014, a total of 1528 ADHF patients were enrolled in the Heart Failure Center of Beijing Fuwai Hospital for sST2 measurement, a prospective study [21]. The result has shown that the population with the highest sST2 concentration quartile (55.6 ng/ml) has a significantly higher rate of adverse outcomes (all-cause mortality or heart transplantation) than patients with the lowest sST2 concentration quartile (25.2 ng/ml).

sST2 concentration correlates with left ventricular ejection fraction. Most patients with reducing LVEF contain higher sST2 concentration, with threshold value dependent on different studies. Results from a global multicenter ADHF cohort study which contains 11 countries [22] show that sST2 concentration has incremental value on traditional clinical variables in predicting 30-day and 1-year mortality at risk stratification. The ASCEND Heart Failure Trial, a multicountry, multicenter prospective randomized controlled trial in ADFF patients [23], conducted marker research for 858 patients from most North American subjects. With a mean LVEF level of 31.6 ± 15%, the ASCEND study found that a higher sST2 concentration is positively associated with the risk of mortality within 180 days with a quarterly sST2 level. However, the prognostic value of baseline sST2 was reduced after adjustment for clinical covariates and NT-proBNP. Also, patients with increasing sST2 concentration (>60 ng/ml) were reported to have higher 180-day mortality than patients with lower sST2 during the follow-up period. Pascual-Figal et al. [24] conducted a prospective study on 107 ADHF inpatients (LVEF 47 ± 15%), and results show that sST2 concentration > 65 ng/ml has a strong predictive value on risk of mortality.

sST2 (reflecting myocardial fibrosis and remodeling), hsTnT (indicating myocardial necrosis), and NT-proBNP (identifying myocardial stretch) concentration provide additional prognostic information for patients with ADHF, respectively. When used together, these markers further provide better risk stratification. sST2 concentration changed rapidly in HF circumstance, especially after diagnosis treatment. When baseline sST2 was validated to have the prognostic effect, more current studies claim that continuous measurements greatly increase the amount of available prognostic information, and the use of sST2 as a monitoring tool appears to be particularly advantageous [25]. In ADHF patients, sST2 concentration tends to decrease after the start of treatment and presents itself as an important prognostic factor both at admission and during hospitalization. Therefore, for ADHF patients, sST2 concentration should be assessed at baseline (for initial risk assessment and shunt) and also measured posttreatment to reflect treatment effectiveness and help decision-making for further treatment.

sST2 also has important prognostic assessment value for CHF patients to help with targeted treatment and follow-up, which potentially improve quality of life and long-term outcomes. The association between sST2 levels and risk of death was determined at the University of Pennsylvania Hospital in a multicenter prospective cohort study (PHFS) which included 1141 CHF outpatients. According to tertiles of sST2 levels, sST2 > 36.3 ng/ml contains higher risk of negative outcomes compared to sST2 ≤ 22.3 ng/ml. sST2 is a powerful biomarker for CHF patients' prognostic assessment, and the interaction between sST2 and NT-proBNP further improves the assessment of the prognosis on top of clinical risk assessment [26]. A German study of 891 outpatients who received treatment for HF showed that in a multivariate Cox proportional risk model, sST2 and NT-proBNP could both significantly predict the risk of mortality [27]. Based on the results of the CORONA study of 1449 patients aged over 60 years with ischemic etiology of CHF (LVEF 40%) [28], the mean sST2 level is 17.8 (IQR: 0.2-400.0 ng/ml). According to COX regression analysis, sST2 > 28.8 ng/ml was found to be associated with CVD mortality, nonfatal MI, and stroke. In 2331 CHF patients (left ventricular ejection fraction < 35%) in the US HF-ACTION study with a median baseline sST2 level of 23.7 ng/ml (IQR: 18.6-31.8) [29], the sST2 level was found to be independently associated with HF patients' prognosis in the multivariate model after adjustment for covariates and NT-proBNP. A doubling of the baseline sST2 level significantly increased the risk of CVD mortality, all-cause mortality, or hospitalization. However, sST2 did not significantly increase the reclassification of risk through the change of *C* statistic, improvement of the net weight classification, and comprehensive discrimination. Despite robust relationships in traditional multivariate modeling, the effect of sST2 on identification and risk prediction is not significant. Further studies are required to illustrate the role of sST2 in CHF risk stratification.

Information about the aforementioned studies about sST2 is recapitulated in Table 1. Despite limited value in diagnosing ADHF and CHF, sST2 have shown vital significance regarding the prognostic process. Increasing studies not only have shown that higher baseline sST2 concentration is frequently associated with a higher risk of adverse event outcomes and mortality but also revealed an additive value of sST2 in the prognosis of ADFH and CHF to traditional predictors; we therefore believe that such markers have great potential and should gain more attention in the clinical practice.

### 3.2. Lp-PLA2

Studies related to Lp-PLA2 have focused on cardiovascular and cerebrovascular diseases due to the proinflammatory and proatherosclerotic effects of Lp-PLA2. Total plasma Lp-PLA2 was found to be predictive in cardiac death in 524 Athens patients with continuous stable coronary artery disease (CAD) who were followed for an average of 34 months [30]. Among 224 African Americans and 336 Caucasians who underwent coronary angiography, Lp-PLA2 activity and index (a comprehensive measure of mass and activity) were determined to be associated with the presence of coronary heart disease (CAD). The findings suggested an independent influence of vascular inflammation in African Americans as a cause of CAD risk and underscored the importance of Lp-PLA2 as a cardiovascular risk factor [31]. Similarly, Lp-PLA2 was found to corelate with CVD events in a nested case-control study based on people with high insulin resistance and diabetes from American Indians [32]. In addition, this study found that the quality of Lp-PLA2 is negatively associated with CVDs. Another study involving 25 isolated patients with isolated coronary artery ectasia (CAE) without stenosis and 25 control groups with normal coronary angiography in the Turkish population found that the Lp-PLA2 level was significantly higher in isolated CAE patients compared with patients with normal angiographic coronary arteries, which suggested that Lp-PLA2 may also be involved in the pathogenesis of CAE [33]. In 4537 US individuals without baseline peripheral artery disease (PAD), higher Lp-PLA2 quality and activity were associated with the occurrence of clinical PAD events [34]. Lp-PLA2 activity was significantly and positively associated with carotid intima-media thickness (IMT) and plaques in 929 Japanese men aged 50-79 years, but the Mendelian randomized study did not support Lp-PLA2 as a cause of subclinical atherosclerosis [35]. Increased Lp-PLA2 activity was found to be associated with significantly fast progression of aortic stenosis (AS) in 183 patients with mild AS from Quebec, Canada, but no corelationship was found in patients with moderate or severe AS [36]. The Lp-PLA2 activity level was significantly associated with coronary heart disease in men and women with type 2 diabetes [37]. However, there are studies that showed opposite results. No significant association between microcerebral hemorrhage (CMB) and Lp-PLA2 measurements was observed in 819 Framingham Offspring participants with an average age of 73 years [38]. A cross-sectional study of 921 nonstroke patients in Barcelona found that although Lp-PLA2 was independently associated with silent cerebral infarction in women, the addition of Lp-PLA2 to clinical variables did not improve its predictive ability [39]. The inconsistent result was observed in studies with the Chinese population as well. Most studies showed that Lp-PLA2 is a risk factor associated with cardiovascular and cerebrovascular diseases. The quality of Lp-PLA2 is positively associated with subclinical atherosclerosis determined by asymptomatic cerebral artery stenosis (ACAS), intracranial artery stenosis (ICAS), and extracranial artery stenosis (ECAS) in north China regions, especially for male gender and older population, which substantiated that Lp-PLA2 quality may be a potential biomarker for the detection of adult ACAS [40]. A prospective study examined Lp-PLA2 quality in 3401 participants from the Chinese Acute Ischemic Stroke Blood Pressure Reduction Trial and found that the accumulated incidence rate for all-cause mortality increased in the quarters of Lp-PLA2 quality [41]. In a multicenter, randomized control clinical trial, Lp-PLA2 activity was not found to increase in patients who were without intracranial artery stenosis, which suggests a better response to dual antiplatelet therapy in patients with mild stroke or high-risk transient ischemic attack to prevent dual stroke and concurrent vascular events [42]. From another Chinese RCT study, a higher level of Lp-PLA2 activity in the onset of the acute phase was associated with an increased risk of short-term recurrent vascular events [43]. However, opposite results have been observed as well. In a 7-year follow-up study that included 90,000 Chinese population, reduction in Lp-PLA2 activity was not associated with a major risk of vascular or nonvascular disease in adults [44]. To sum up, controversy about the role of Lp-PLA2 in cardiovascular and cerebrovascular diseases does exist because of inconsistent and limited studies. More researches with diverse data characteristics and huge data sizes should be developed to validate the practicability of Lp-PLA2.

### 3.3. MPO

There is elevated evidence shown that circulation MPO protein concentration is not only associated with the rate of coronary artery disease (CAD) but also related to the severity of the disease. A case-control study including 874 angiographically proven CAD patients has shown an advanced MPO level in CAD patients and elevated MPO level in the progress of non-ST segment elevation of the acute coronary syndrome and acute myocardial infarction from stable CAD [45]. The study by Samsamshariat et al. included 50 cases of stable CAD patients, unstable CAD patients, and the control group, respectively. Its results showed that unstable CAD patients had a higher level of plasma MPO level (71.2 ± 19.6 ng/ml) than both stable CAD patients (34.5 ± 6.8 ng/ml) and control groups (23.0 ± 3.6 ng/ml) (*P* < 0.001) [46]. Study from Pawlus et al. and Tretjakovs et al. reported that serum MPO concentration increased with the development of CAD, and patients with unstable CAD and myocardial infarction have a significantly elevated level of MPO compared to healthy subjects and stable angina pectoris patients [47, 48].

Furthermore, in patients who are diagnosed with acute myocardial infarction, the MPO level is higher than patients with angina pectoris [49, 50] or stable CAD [51]; meanwhile, there is a significant association between MPO concentration and angiographically detected coronary artery stenosis [52, 53]. Rebeiz et al. reported that elevation in plasma MPO could be used to identify coronary artery stenosis in 398 cases of negative chest pain with troponin patients, in which elevated quartiles of MPO concentration are closely associated with coronary artery stenosis, coronary thrombosis, and plaque ulcers [54]. It is noteworthy that, for patients who suffered from fatal or nonfatal CAD, the aforementioned relationship is much stronger, which confirmed the idea that the MPO level is associated with the severity of CAD. Overall, a large body of studies has supported the association between elevated MPO levels with CAD and a dose-response relationship between MPO levels and the severity of CAD.

Some studies revealed the predictive ability of MPO on major adverse cardiovascular events (MACE). In 604 chest pain patients collected by Brennan et al., an increase in the MPO level was independently associated with advanced risk of MACE at 30 days and 6 months of follow-up after adjustment for risk factors [55]. More specifically, at 30 days, cardiac troponin alone can predict 58% of MACE, while baseline MPO is able to predict 84.5% of MACE. MPO remained a strong and independent predictor of MACE at 30 days and 6 months in the patients who were consistently troponin-negative. This suggested that MPO is not only an inflammatory marker of myocardial necrosis response but also a predictor of the presence of susceptible plaques. Brennan et al.'s study showed that plasma MPO could independently predict the early stage of acute myocardial infarction and the risk of MACE in the period of 30 days and 6 months, meanwhile also forehead its potential practicability on the risk stratification of chest pain patients [55]. Similarly, the CAPTURE trial, which involved 547 patients with resting recurrent chest pain, observed that MPO serum levels of >350 ng/ml were highly predictive of nonfatal acute myocardial infarction within 72 hours [56]. Such corelationship remained robust even in patients who are troponin-negative. At the same time, Morrow et al. claimed that elevated baseline MPO in patients with non-ST-elevation ACS was associated with a higher risk of nonfatal MI or recurrent ACS within 30 days, and the addition of B-type natriuretic peptides and cardiac troponin to the MPO can improve short-term risk assessment and patient stratification [57].

Some studies also found that such predictive value may persist over a long period of time. In a research with a mean follow-up time of 25 ± 16 months, 73 patients diagnosed as ST segment elevation myocardial infarction subjects and 46 health control groups and patients with plasma MPO concentration > 68 ng/ml have tripled the incident rate of MACE compared to patients with low MPO [58]. Among 885 stable severe angiography CAD patients, mortality is positively associated with MPO tertiles. After 13 years of follow-up and covariate analysis, patients with the highest MPO have as much as three times greater CVD mortality than those with the lowest MPO level [53]. During 9 years of angiographic CAD studies, patients with the highest MPO quartile were 1.5 times more likely to die of cardiovascular causes than patients with the lowest MPO quartile after fully adjusting for variables [59]. These findings suggested that from a mid- to long-term perspective, MPO is highly associated with cardiovascular events.

A prospective population-based Atherosclerosis Risk in Communities (ARIC) cohort study, after 9.6 years of the follow-up study, found that no significant association between intracellular monocyte MPO level and incident of cardiovascular events was found among 1465 patients without a history of PAD, myocardial infarction, or heart failure [60].

The MPO level is independently associated with cardiovascular events, and the dose-dependent relationship between MPO level and CAD severity also has great potential in guiding the diagnostic and treatment process in clinical practice. However, the prediction value of MPO on MACE in asymptomatic patients has remained uncertain and needs to be explored in more and larger studies.

### 3.4. PCT

It is difficult to distinguish acute heart failure (AHF) from respiratory infection for patients with HF with acute respiratory symptoms (e.g., cough, sputum, tachypnea, or pleural pain) [61] due to the overlapping clinical and radiological examination [62, 63]. Delayed symptomatic treatment [64] or inadequate treatment [65] can increase the risk of adverse outcomes, so on-time and accurate differential diagnosis is crucial in which field.

In a study consisting of 4698 patients with congestive heart failure, PCT value was found about 4 times higher in patients with HF with the presence of respiratory infections [66]. Similar results have been found in biomarkers for heart failure research in a different country [65]. At present, most studies have shown that serum PCT has certain clinical significance in the diagnosis of patients with heart failure complicated with infection. Berge et al. believed that PCT is superior to CRP and WBC when it comes to the diagnosis of AHF patients for concomitant pneumonia [67]. A large multicenter study in China was conducted with patients with congestive HF, and the results showed that the critical value of PCT for the diagnosis of infection increased significantly with the increase of the severity of HF, and the threshold value of PCT for the diagnosis of concomitant infection in patients with grades II, III, and IV congestive HF is 0.086 *μ*g/l, 0.0192 *μ*g/l, and 0.657 *μ*g/l [66]. Alba et al.'s research claims that the cutoff PCT value for pneumonia excluding heart failure is 0.1 ng/ml (sensitivity 97%, specificity 69%) and for HF with pneumonia is 0.4 ng/ml (specificity 97%) and believed that the combined use of PCT and NT-proBNP can more effectively distinguish AHF and pneumonia [68]. In short, it can be seen that PCT can be helpful in determining respiratory tract infection, but the inconsistency in threshold value suggested further researchers.

Some studies have observed the correlation between PCT and bacteremia, which is the primary diagnostic criterion for endocarditis in emergency department patients with fever [69, 70]. In an observational study involving 1083 patients with suspected infection, PCT was strongly associated with positive blood culture (AUC 0.803) and has a negative predictive value of 99.6% in bacteriaemia exclusion [70]. A prospective cohort study which consisted of 67 consecutive admissions with suspected endocarditis and 21 patients with an infection found a strong association between PCT levels with endocarditis and significantly higher procalcitonin levels in patients with endocarditis (median 6.56 ng/ml vs. 0.44 ng/ml, *P* < 0.001, AUC 0.856 (95% CI 0.750-0.962)). In this study, the sensitivity and predictive value of PCT also outperformed the CRP, with the optima PCT cutoff value of 2.3 ng/ml, sensitivity 81%, specificity 85%, negative predictive value 92%, and positive predictive value 72% [71]. A recent meta-analysis reviewed six studies, including more than 1000 suspected cases of infection, of which about 20% were confirmed as infective endocarditis, showing a slightly lower diagnostic effect of PCT (AUC 0.71) [72]. Meanwhile, this meta-analysis suggested the use of a low PCT threshold to exclude endocarditis in routine clinical practice, and PCT is recommended in the diagnosis of infective endocarditis, but the specific threshold value needs to be further explored.

In order to study the clinical significance of PCT in the prognosis of AHF patients, Demissei et al. included 1781 patients with acute decompensated heart failure (ADHF) excluding those with infection from the PROTECT research to investigate the corelationship between outcomes during hospitalization and after discharge with the serum PCT level [73]. In this study, an elevated PCT level was found to be associated with an increased rate of treatment failure in ADHF patients during hospitalization (OR 1.2, 95% CI, 0.8-2.2). When the PCT level > 0.2 ng/ml, cardiovascular, renal mortality, or readmission rate at 30 days after discharge and all-cause mortality at 180 days after discharge both significantly increased. However, after adjusting for prognostic factors including age, albumin, sodium, renal function, previous HF hospitalization, history of edema, and systolic blood pressure, the PCT level in ADHF patients with the exclusion of infection is only associated with 30-day all-cause mortality (HR 2.3, 95% CI, 1.3-4.2).

In Villanueva et al.'s research on 261 AHF patients with the exclusion of active infection, after a mean follow-up time of 2 years, logPCT was found to be linearly associated with all-cause mortality in COX regression analysis (HR 1.43, 95% CI, 1.12-1.82, *P* = 0.0004). LogPct was found to be positively correlated with the incidence of all-cause readmissions (IRR = 1.22, 95% CI, 1.02-1.44, *P* = 0.025), except for patients who were lost during follow-up (died during admission), and also associated with AHF-induced readmission (IRR = 1.28, 95% CI, 1.02-1.61, *P* = 0.032). This study concluded that the serum PCT level in AHF patients with infection excluded was independently positively associated with the risk of long-term death and rehospitalization.

In addition to its predictive value in the prognosis of patients with heart failure, there is growing evidence linking PCT with the degree and prognosis of atherosclerosis in patients with coronary heart disease. More studies have shown that in patients with acute coronary syndrome, especially in complex cases, the PCT level is elevated [74, 75]. Research conducted on 2131 cases of CAD patients found that PCT has a strong prognostic value after 3.6 years of follow-up [74]. PCT concentration was significantly higher in patients who died of cardiovascular diseases, as well as in patients with acute coronary syndromes, compared with patients with stable angina. In the COX regression model, elevated PCT concentration is associated with cardiovascular mortality (OR 1.34, 95% CI, 1.08-1.65, *P* = 0.007). In another research that included 400 cases of CAD patients with the SX scoring algorithm system, the high SX group have elevated serum PCT levels compared to the low SX group (*P* < 0.001) [76]. The cutoff value for PCT in the aforementioned study is 0.0335 ng/ml.

The detection of PCT will be helpful in the diagnosis and treatment of patients with bacterial infection and endocarditis. Moreover, for patients without infection, a high PCT level is an independent risk factor for poor prognosis.

## 4. Conclusion

Increasing studies have focused on exploring the synergy value or independent value of biomarkers, while the limitations of currently widely used blood markers have been regularly reported in clinical practice. The current study reviewed some frequently overlooked biomarkers that have shown significance in both diagnostic and prognostic processes. Despite accumulating evidence suggesting their association with CVDs and their predictive ability on diseases' prognostic process, more research is needed to establish the clinical role of biomarkers.

## Figures and Tables

**Table 1:** Summary of clinical studies on the relationship between sST2 and adverse outcomes.

Study	Country	Patients	Outcome	sST2 measurement method	sST2 concentration for higher risk
PRIDE study [20]	USA	593 patients with acute dyspnea	1-year mortality	Enzyme-linked immunosorbent assay	67.4 vs. 35.8 ng/ml
			4-year mortality	Enzyme-linked immunosorbent assay	47.4 vs. 35.6 ng/ml
Heart Failure Center of Beijing Fuwai Hospital [21]	China	1528 ADHF patients	Adverse outcomes (all-cause mortality or heart transplantation)		≥55.6 ng/ml vs. <25.2 ng/ml
MOCA study [22]	11 countries^∗^	5306 patients hospitalized for ADHF	30-day and 1-year mortality	—	—
ASCEND Heart Failure Trial [23]	USA	858 acute HF subjects with a mean LVEF level of 31.6 ± 15%	180-day mortality	The presage ST2 assay	>60 ng/ml
University Hospital Virgen de la Arrixaca [24]	Spain	107 consecutive ADHF patients	All-cause mortality	High-sensitivity sandwich immunoassay	>65 ng/ml
Penn Heart Failure Study (PHFS) [26]	USA	1141 CHF outpatients	All-cause mortality or cardiac transplantation	Highly sensitive sandwich monoclonal immunoassay	>36.3 ng/ml vs. ≤22.3 ng/ml
A German study by Bayes-Genis et al. [27]	Spain	891 ambulatory patients	All-cause mortality	High-sensitivity sandwich monoclonal immunoassay	>50 ng/ml
CORONA study [28]	Multicountry	1449 patients aged over 60 years with ischemic etiology of CHF (LVEF 40%)	CVD mortality, nonfatal MI, and stroke	Presage® ST2 assay	>28.8 ng/ml
HF-ACTION study [29]	Greece	2331 CHF patients (LVEF < 35%)	All-cause mortality or hospitalization, cardiovascular mortality or heart failure hospitalization, and all-cause mortality	Dual monoclonal antibody immunoassay	>35 ng/ml

^∗^Austria (*n* = 137), Czech Republic (*n* = 1917), Finland (*n* = 620), France (*n* = 199), Netherlands (*n* = 367), Italy (*n* = 213), Japan (*n* = 144), Spain (*n* = 107), Switzerland (*n* = 609), Tunisia (*n* = 187), and the United States (*n* = 597 for Cleveland, *n* = 209 for Boston).

## Data Availability

The data supporting this review are from previously reported studies and datasets, which have been cited at relevant places within the text as References [1–75].

## References

[1] GBD 2013 DALYs and HALE Collaborators, Murray C. J. L., Barber R. M. (2015). Global, regional, and national disability-adjusted life years (DALYs) for 306 diseases and injuries and healthy life expectancy (HALE) for 188 countries, 1990-2013: quantifying the epidemiological transition. *Lancet*.

[2] Iwahana H., Yanagisawa K., Ito-Kosaka A. (1999). Different promoter usage and multiple transcription initiation sites of the interleukin-1 receptor-related human ST2 gene in UT-7 and TM12 cells. *European Journal of Biochemistry*.

[3] Kakkar R., Lee R. T. (2008). The IL-33/ST2 pathway: therapeutic target and novel biomarker. *Nature Reviews. Drug Discovery*.

[4] Weinberg E. O., Shimpo M., de Keulenaer G. W. (2002). Expression and regulation of ST2, an interleukin-1 receptor family member, in cardiomyocytes and myocardial infarction. *Circulation*.

[5] Huang F., Wang K., Shen J. (2020). Lipoprotein-associated phospholipase A2: the story continues. *Medicinal Research Reviews*.

[6] Jensen M. K., Bertoia M. L., Cahill L. E., Agarwal I., Rimm E. B., Mukamal K. J. (2014). Novel metabolic biomarkers of cardiovascular disease. *Nature Reviews. Endocrinology*.

[7] Davies M. J., Hawkins C. L. (2020). The role of myeloperoxidase in biomolecule modification, chronic inflammation, and disease. *Antioxidants & Redox Signaling*.

[8] Kettle A. J., Anderson R. F., Hampton M. B., Winterbourn C. C. (2007). Reactions of superoxide with myeloperoxidase. *Biochemistry*.

[9] Teng N., Maghzal G. J., Talib J., Rashid I., Lau A. K., Stocker R. (2017). The roles of myeloperoxidase in coronary artery disease and its potential implication in plaque rupture. *Redox Report*.

[10] Emerging Risk Factors Collaboration, di Angelantonio E., Sarwar N. (2009). Major lipids, apolipoproteins, and risk of vascular disease. *JAMA*.

[11] Liu W. Q., Zhang Y. Z., Wu Y. (2015). Myeloperoxidase-derived hypochlorous acid promotes ox-LDL-induced senescence of endothelial cells through a mechanism involving *β*-catenin signaling in hyperlipidemia. *Biochemical and Biophysical Research Communications*.

[12] Koeth R. A., Haselden V., Tang W. H. (2013). Myeloperoxidase in cardiovascular disease. *Advances in Clinical Chemistry*.

[13] Stocker R., Huang A., Jeranian E. (2004). Hypochlorous acid impairs endothelium-derived nitric oxide bioactivity through a superoxide-dependent mechanism. *Arteriosclerosis, Thrombosis, and Vascular Biology*.

[14] Picariello C., Lazzeri C., Valente S., Chiostri M., Gensini G. F. (2011). Procalcitonin in acute cardiac patients. *Internal and Emergency Medicine*.

[15] Muller B., White J. C., Nylen E. S., Snider R. H., Becker K. L., Habener J. F. (2001). Ubiquitous expression of the calcitonin-i gene in multiple tissues in response to sepsis. *The Journal of Clinical Endocrinology and Metabolism*.

[16] Linscheid P., Seboek D., Zulewski H., Keller U., Muller B. (2005). Autocrine/paracrine role of inflammation-mediated calcitonin gene-related peptide and adrenomedullin expression in human adipose tissue. *Endocrinology*.

[17] Christ-Crain M., Muller B. (2005). Procalcitonin in bacterial infections--hype, hope, more or less?. *Swiss Medical Weekly*.

[18] Schuetz P., Daniels L. B., Kulkarni P., Anker S. D., Mueller B. (2016). Procalcitonin: a new biomarker for the cardiologist. *International Journal of Cardiology*.

[19] Januzzi J. L., Peacock W. F., Maisel A. S. (2007). Measurement of the interleukin family member ST2 in patients with acute dyspnea: results from the PRIDE (Pro-Brain Natriuretic Peptide Investigation of Dyspnea in the Emergency Department) study. *Journal of the American College of Cardiology*.

[20] Januzzi J. L., Mebazaa A., Di Somma S. (2015). ST2 and prognosis in acutely decompensated heart failure: the International ST2 Consensus Panel. *The American Journal of Cardiology*.

[21] Zhang R., Zhang Y., Zhang J. (2014). The prognostic value of plasma soluble ST2 in hospitalized Chinese patients with heart failure. *PLoS One*.

[22] Lassus J., Gayat E., Mueller C. (2013). Incremental value of biomarkers to clinical variables for mortality prediction in acutely decompensated heart failure: the Multinational Observational Cohort on Acute Heart Failure (MOCA) study. *International Journal of Cardiology*.

[23] Tang W. H., Wu Y., Grodin J. L. (2016). Prognostic value of baseline and changes in circulating soluble ST2 levels and the effects of nesiritide in acute decompensated heart failure. *JACC Heart Fail*.

[24] Pascual-Figal D. A., Manzano-Fernández S., Boronat M. (2011). Soluble ST2, high-sensitivity troponin T- and N-terminal pro-B-type natriuretic peptide: complementary role for risk stratification in acutely decompensated heart failure. *European Journal of Heart Failure*.

[25] Manzano-Fernández S., Januzzi J. L., Pastor-Pérez F. J. (2012). Serial monitoring of soluble interleukin family member ST2 in patients with acutely decompensated heart failure. *Cardiology*.

[26] Ky B., French B., McCloskey K. (2011). High-sensitivity ST2 for prediction of adverse outcomes in chronic heart failure. *Circulation. Heart Failure*.

[27] Bayes-Genis A., de Antonio M., Galán A. (2012). Combined use of high-sensitivity ST2 and NTproBNP to improve the prediction of death in heart failure. *European Journal of Heart Failure*.

[28] Broch K., Ueland T., Nymo S. H. (2012). Soluble ST2 is associated with adverse outcome in patients with heart failure of ischaemic aetiology. *European Journal of Heart Failure*.

[29] Felker G. M., Fiuzat M., Thompson V. (2013). Soluble ST2 in ambulatory patients with heart failure: association with functional capacity and long-term outcomes. *Circulation. Heart Failure*.

[30] Rallidis L. S., Tellis C. C., Lekakis J. (2012). Lipoprotein-Associated Phospholipase A_2_ Bound on High- Density Lipoprotein Is Associated With Lower Risk for Cardiac Death in Stable Coronary Artery Disease Patients: A 3-Year Follow-Up. *Journal of the American College of Cardiology*.

[31] Anuurad E., Ozturk Z., Enkhmaa B., Pearson T. A., Berglund L. (2010). Association of lipoprotein-associated phospholipase A2 with coronary artery disease in African-Americans and Caucasians. *The Journal of Clinical Endocrinology and Metabolism*.

[32] Kizer J. R., Umans J. G., Zhu J. (2012). Lipoprotein-associated phospholipase A(2) mass and activity and risk of cardiovascular disease in a population with high prevalences of obesity and diabetes: the Strong Heart Study. *Diabetes Care*.

[33] Korkmaz L., Erkuş E., Kırış A. (2011). Lipoprotein phospholipase A2 in patients with isolated coronary artery ectasia. *Clinical Research in Cardiology*.

[34] Garg P. K., Arnold A. M., Hinckley Stukovsky K. D. (2016). Lipoprotein-associated phospholipase A2 and incident peripheral arterial disease in older adults: the Cardiovascular Health Study. *Arteriosclerosis, Thrombosis, and Vascular Biology*.

[35] Ueshima H., Kadowaki T., Hisamatsu T. (2016). Lipoprotein-associated phospholipase A_2_ is related to risk of subclinical atherosclerosis but is not supported by Mendelian randomization analysis in a general Japanese population. *Atherosclerosis*.

[36] Capoulade R., Mahmut A., Tastet L. (2015). Impact of plasma Lp-PLA2 activity on the progression of aortic stenosis: the PROGRESSA study. *JACC: Cardiovascular Imaging*.

[37] Hatoum I. J., Hu F. B., Nelson J. J., Rimm E. B. (2010). Lipoprotein-associated phospholipase A2 activity and incident coronary heart disease among men and women with type 2 diabetes. *Diabetes*.

[38] Romero J. R., Preis S. R., Beiser A. S. (2012). Lipoprotein phospholipase A2 and cerebral microbleeds in the Framingham Heart Study. *Stroke*.

[39] Riba-Llena I., Penalba A., Pelegrí D. (2014). Role of lipoprotein-associated phospholipase A_2_ activity for the prediction of silent brain infarcts in women. *Atherosclerosis*.

[40] Nazerian P., Mueller C., Soeiro A. M. (2018). Diagnostic accuracy of the aortic dissection detection risk score plus D-dimer for acute aortic syndromes: the ADvISED prospective multicenter study. *Circulation*.

[41] Han L., Zhong C., Bu X. (2017). Prognostic value of lipoprotein-associated phospholipase A_2_ mass for all-cause mortality and vascular events within one year after acute ischemic stroke. *Atherosclerosis*.

[42] Yang M., Wang A., Li J. (2020). Lp-PLA2and dual antiplatelet agents in intracranial arterial stenosis. *Neurology*.

[43] Lin J., Zheng H., Cucchiara B. L. (2015). Association of Lp-PLA2-A and early recurrence of vascular events after TIA and minor stroke. *Neurology*.

[44] Millwood I. Y., Bennett D. A., Walters R. G. (2016). A phenome-wide association study of a lipoprotein-associated phospholipase A2 loss-of-function variant in 90 000 Chinese adults. *International Journal of Epidemiology*.

[45] Ndrepepa G., Braun S., Mehilli J., von Beckerath N., Schomig A., Kastrati A. (2008). Myeloperoxidase level in patients with stable coronary artery disease and acute coronary syndromes. *European Journal of Clinical Investigation*.

[46] Samsamshariat S. Z., Basati G., Movahedian A., Pourfarzam M., Sarrafzadegan N. (2011). Elevated plasma myeloperoxidase levels in relation to circulating inflammatory markers in coronary artery disease. *Biomarkers in Medicine*.

[47] Pawlus J., Holub M., Kozuch M., Dabrowska M., Dobrzycki S. (2010). Serum myeloperoxidase levels and platelet activation parameters as diagnostic and prognostic markers in the course of coronary disease. *International Journal of Laboratory Hematology*.

[48] Tretjakovs P., Jurka A., Bormane I. (2012). Circulating adhesion molecules, matrix metalloproteinase-9, plasminogen activator inhibitor-1, and myeloperoxidase in coronary artery disease patients with stable and unstable angina. *Clinica Chimica Acta*.

[49] Khan D. A., Sharif M. S., Khan F. A. (2011). Diagnostic performance of high-sensitivity troponin T, myeloperoxidase, and pregnancy-associated plasma protein A assays for triage of patients with acute myocardial infarction. *The Korean Journal of Laboratory Medicine*.

[50] Trentini A., Rosta V., Spadaro S. (2020). Development, optimization and validation of an absolute specific assay for active myeloperoxidase (MPO) and its application in a clinical context: role of MPO specific activity in coronary artery disease. *Clinical Chemistry and Laboratory Medicine*.

[51] Goldmann B. U., Rudolph V., Rudolph T. K. (2009). Neutrophil activation precedes myocardial injury in patients with acute myocardial infarction. *Free Radical Biology & Medicine*.

[52] Baseri M., Heidari R., Mahaki B., Hajizadeh Y., Momenizadeh A., Sadeghi M. (2014). Myeloperoxidase levels predicts angiographic severity of coronary artery disease in patients with chronic stable angina. *Advanced Biomedical Research*.

[53] Heslop C. L., Frohlich J. J., Hill J. S. (2010). Myeloperoxidase and C-reactive protein have combined utility for long-term prediction of cardiovascular mortality after coronary angiography. *Journal of the American College of Cardiology*.

[54] Rebeiz A. G., Tamim H. M., Sleiman R. M. (2011). Plasma myeloperoxidase concentration predicts the presence and severity of coronary disease in patients with chest pain and negative troponin-T. *Coronary Artery Disease*.

[55] Brennan M. L., Penn M. S., van Lente F. (2003). Prognostic value of myeloperoxidase in patients with chest pain. *The New England Journal of Medicine*.

[56] Baldus S., Heeschen C., Meinertz T. (2003). Myeloperoxidase serum levels predict risk in patients with acute coronary syndromes. *Circulation*.

[57] Morrow D. A., Sabatine M. S., Brennan M. L. (2008). Concurrent evaluation of novel cardiac biomarkers in acute coronary syndrome: myeloperoxidase and soluble CD40 ligand and the risk of recurrent ischaemic events in TACTICS-TIMI 18. *European Heart Journal*.

[58] Kaya M. G., Yalcin R., Okyay K. (2012). Potential role of plasma myeloperoxidase level in predicting long-term outcome of acute myocardial infarction. *Texas Heart Institute Journal*.

[59] Scharnagl H., Kleber M. E., Genser B. (2014). Association of myeloperoxidase with total and cardiovascular mortality in individuals undergoing coronary angiography--the LURIC study. *International Journal of Cardiology*.

[60] Oyenuga A. O., Couper D., Matsushita K., Boerwinkle E., Folsom A. R. (2018). Association of monocyte myeloperoxidase with incident cardiovascular disease: the Atherosclerosis Risk in Communities Study. *PLoS One*.

[61] Maisel A., Mueller C., Nowak R. (2010). Mid-region pro-hormone markers for diagnosis and prognosis in acute dyspnea: results from the BACH (Biomarkers in Acute Heart Failure) trial. *Journal of the American College of Cardiology*.

[62] Nanbu T., Satou I., Nishijima H., Kitabatake A. (1997). Differentiation of vasospastic angina from noncardiac chest pain by history and coronary risk factors in patients with chest pain at rest. *Internal Medicine*.

[63] Tsuyuki R. T., McKelvie R. S., Arnold J. M. (2001). Acute precipitants of congestive heart failure exacerbations. *Archives of Internal Medicine*.

[64] Wuerz R. C., Meador S. A. (1992). Effects of prehospital medications on mortality and length of stay in congestive heart failure. *Annals of Emergency Medicine*.

[65] Maisel A., Neath S. X., Landsberg J. (2012). Use of procalcitonin for the diagnosis of pneumonia in patients presenting with a chief complaint of dyspnoea: results from the BACH (Biomarkers in Acute Heart Failure) trial. *European Journal of Heart Failure*.

[66] Wang W., Zhang X., Ge N. (2014). Procalcitonin testing for diagnosis and short-term prognosis in bacterial infection complicated by congestive heart failure: a multicenter analysis of 4,698 cases. *Critical Care*.

[67] Berge K., Lyngbakken M. N., Einvik G. (2018). Diagnostic and prognostic properties of procalcitonin in patients with acute dyspnea: data from the ACE 2 study. *Clinical Biochemistry*.

[68] Alba G. A., Truong Q. A., Gaggin H. K. (2016). Diagnostic and prognostic utility of procalcitonin in patients presenting to the emergency department with dyspnea. *The American Journal of Medicine*.

[69] Rast A. C., Kutz A., Felder S. (2015). Procalcitonin improves the Glasgow prognostic score for outcome prediction in emergency patients with cancer: a cohort study. *Disease Markers*.

[70] Laukemann S., Kasper N., Kulkarni P. (2015). Can we reduce negative blood cultures with clinical scores and blood markers? Results from an observational cohort study. *Medicine (Baltimore)*.

[71] Mueller C., Huber P., Laifer G., Mueller B., Perruchoud A. P. (2004). Procalcitonin and the early diagnosis of infective endocarditis. *Circulation*.

[72] Yu C. W., Juan L. I., Hsu S. C. (2013). Role of procalcitonin in the diagnosis of infective endocarditis: a meta- analysis. *The American Journal of Emergency Medicine*.

[73] Demissei B. G., Cleland J. G., O'Connor C. M. (2016). Procalcitonin-based indication of bacterial infection identifies high risk acute heart failure patients. *International Journal of Cardiology*.

[74] Sinning C. R., Sinning J. M., Schulz A. (2011). Association of serum procalcitonin with cardiovascular prognosis in coronary artery disease. *Circulation Journal*.

[75] Remskar M., Horvat M., Hojker S., Noc M. (2002). Procalcitonin in patients with acute myocardial infarction. *Wiener Klinische Wochenschrift*.

[76] Kurtul A., Elcik D. (2017). Procalcitonin is an independent predictor for coronary atherosclerotic burden in patients with stable coronary artery disease. *International Journal of Cardiology*.

